# Ilm-NMR-P31: an open-access ^31^P nuclear magnetic resonance database and data-driven prediction of ^31^P NMR shifts

**DOI:** 10.1186/s13321-023-00792-y

**Published:** 2023-12-18

**Authors:** Jasmin Hack, Moritz Jordan, Alina Schmitt, Melissa Raru, Hannes Sönke Zorn, Alex Seyfarth, Isabel Eulenberger, Robert Geitner

**Affiliations:** Institute of Chemistry and Bioengineering, Group of Physical Chemistry/Catalysis, Technical University Ilmenau, Weimarer Str. 32, 98693 Ilmenau, Germany

**Keywords:** NMR, Phosphorus, Database, Prediction, Increment system, Fingerprint, HOSE, Graph neural network

## Abstract

This publication introduces a novel open-access ^31^P Nuclear Magnetic Resonance (NMR) shift database. With 14,250 entries encompassing 13,730 distinct molecules from 3,648 references, this database offers a comprehensive repository of organic and inorganic compounds. Emphasizing single-phosphorus atom compounds, the database facilitates data mining and machine learning endeavors, particularly in signal prediction and Computer-Assisted Structure Elucidation (CASE) systems. Additionally, the article compares different models for ^31^P NMR shift prediction, showcasing the database’s potential utility. Hierarchically Ordered Spherical Environment (HOSE) code-based models and Graph Neural Networks (GNNs) perform exceptionally well with a mean squared error of 11.9 and 11.4 ppm respectively, achieving accuracy comparable to quantum chemical calculations.

## Introduction

Phosphorus-31 Nuclear Magnetic Resonance (^31^P NMR) spectroscopy is a powerful analytical tool for the characterization of organic, inorganic, and biological compounds. The chemical shift of the ^31^P nucleus is sensitive to the local electronic environment, providing valuable information on the structure, bonding, and reactivity of phosphorus-containing molecules. The interpretation of ^31^P NMR spectra, however, can be challenging, as the chemical shift is influenced by a variety of factors, such as the coordination number, oxidation state, and stereochemistry of the phosphorus atom, as well as the nature and proximity of the neighboring atoms. To facilitate the identification and assignment of ^31^P NMR spectra, several databases have been developed, which compile experimental chemical shift values and corresponding structural information for a range of phosphorus compounds.

The main use of NMR databases has been the training of signal prediction and Computer-Assisted Structure Elucidation (CASE) systems. Traditionally, quantum chemical calculations have been employed to simulate NMR spectra and achieved high accuracy [1–3]. These methods e.g. density functional theory (DFT) based approaches, rely on the description of the electron density distribution to determine local shielding effects for each atom. These shielding constants can later be transformed into NMR spectra. For instance, Payard et al. utilized gauge-independent atomic orbital (GIAO) calculations to predict ^31^P chemical shifts of first row transition metal complexes. They achieved results with a mean absolute error (MAE) of 7.7 ppm [4]. Although quantum mechanical calculations are accurate and have been standard in the field, they are time-consuming and computationally intensive.

In contrast to quantum chemical calculations, machine learning (ML) techniques have gained considerable interest. Several publications have been made, which predict chemical shifts using ML. These techniques, such as hierarchically ordered spherical environment (HOSE), graph neural networks (GNNs) as well as increment and fingerprint models offer promising results in terms of accuracy, speed and ease of use by bypassing quantum mechanical calculations [5–7].

HOSE codes encode a chemical environment around an atom in a molecule in a spherical manner [8]. Hierarchically, the HOSE code moves from the closest to the furthest substituents. Using this method, the code searches databases for the given environment, prioritizing the closest match and predicting the chemical shift [9]. Traditionally, HOSE codes do not include stereochemical information, however there are stereo-aware code extensions, that produce more accurate chemical shift predictions [10]. Furthermore, GNNs are neural networks that operate on graph-based data. In chemistry, molecular structures can be expressed through undirected graphs, in which nodes represent atoms and edges represent bonds. By using entire chemical structures as an input, a GNN studies local representations centered at each atom to predict specific properties, such as chemical shifts [11]. Another technique for the prediction of NMR shifts are increment models. The model assumes that each substituent of a given atom contributes additively to the final chemical shift being predicted. Hence, the chemical shift is calculated as the sum of each substituent’s contribution added to the main chemical shift [12]. Finally, molecular fingerprints can be used to turn molecules into vectors of fixed length, e.g. 881 in case of the MACCS keys, to subsequently use these vectors as descriptors in machine learning applications to predict specific chemical properties. To the best of our knowledge, fingerprint models have not been used to predict chemical shifts as they only allow the prediction of one property per molecule, instead of targeting atom specific properties. Although, this approach is commonly used in other fields, it seems especially promising for the prediction of hetero nuclear NMR signals [13, 14].

An important small-scale study, which does not include quantum chemical calculations, was produced by Tong et al. They predicted ^31^P chemical shifts by modeling the fundamental quantitative structure spectroscopy relationship by analyzing the relationship between the ionicity index and spectroscopy effect parameters for phosphorus atoms in phosphines. They developed a quantitative equation using multiple linear regressions to calculate ^31^P chemical shifts, with a root mean square error (RMSE) of 13.9 ppm [15].

Proprietary software, such as Mestrelab Research’s MNova or ACD/Labs’ NMR Predictor, are noteworthy commercial tools for large scale hetero nuclear NMR shift prediction. Both include ^31^P spectra predictions, are fast and easy to use. Due to the commercial nature of these software solutions, little is known about the inner workings of the prediction models. Both Mestrelab and ACD/Labs stated that they use ensemble prediction algorithms, which include HOSE codes and Neural Networks [16, 17].

In this manuscript, we present a new ^31^P NMR shift database named “Ilm-NMR-P31”, its content, features, limitations, and how it compares to other existing ^31^P NMR databases. The aim of this project is to provide a large open-access ^31^P database for data mining and machine learning projects. As an application, we present, compare, and discuss the prediction of ^31^P NMR shifts using HOSE code, GNNs, increment- and fingerprint-based models.

## Materials and methods

### Database construction

The goal of the project was to digitalize the ^31^P NMR information found in “Numerical Data and Functional Relationships in Science and Technology: NMR Data for Phosphorus-31” (Landolt-Börnstein) from 2014 [18] and in the “Handbook of Phosphorus-31 Nuclear Magnetic Resonance Data” (Handbook of ^31^P NMR) from 1991 [19]. Both tables report the structure as well as the ^31^P shift of small organic and inorganic molecules, while also featuring a small (< 300 molecules) section of biological relevant compounds. Both books are available as PDF documents and could thus partially be read by machines.

The data processing was mainly done using R (4.3.1) [20] and its packages. The table like structure of the Handbook of ^31^P NMR was digitalized using Tabula (1.2.1) [21] which saved the information as CSV files. The tables list the structures as text strings, the ^31^P NMR shift(s), the original reference(s) and occasionally coupling constants, which were not considered for the database build-up. Unfortunately, no information on the solvent or temperature was listed. The data was corrected manually when the optical character recognition (OCR) implemented in Tabula made flawed readouts. This happened regularly with the characters “l” (lowercase “L”) and “1” (one), “O” and “0” (zero) or sub- and superscripted numbers. The regular outline of the Landolt-Börnstein enabled us to extract the NMR shift, solvent and reference information directly form the PDF file using the R packages pdftools [22] and stringr [23]. Information on the infrastructure used to measure the ^31^P spectra, mainly the field strength and manufacturer of the NMR spectrometer were also not listed and are thus also not available in our digital database.

We tried to use optical chemical structure recognition tools [24] to digitalize the structures found in the Landolt-Börnstein, but had erroneous structures with a rate of > 10% which made manual inspection and correction unavoidable. The Handbook of ^31^P NMR displays its structures as text strings and only partially as Lewis formula. As the representation in the Handbook of ^31^P NMR was not standardized and therefore varied between chapters, manual inspection was also necessary. Thus, all structures found in the two books were hand drawn using ACD/ChemSketch (Freeware, 2021.2.0) [25], saved as MOL files and later processed.

The shift data was either read from the CSV files for the Handbook of ^31^P NMR or directly from the PDF for the Landolt-Börnstein. The character strings were whitespace trimmed and subset using the R package stringr [23]. The reference numbers listed for each molecule in the Handbook of ^31^P NMR were replaced by the complete reference given at the end of each chapter.

The MOL files were read into R using the package ChemmineR [26] and further processed using the package ChemmineOB [27] and software OpenBabel (3.1.1) [28]. All hydrogen atoms were made explicit and all molecules were transformed into the SDF format. Based on this SDF information, the sum formulae, the molecular weights, the number of carbon, nitrogen, phosphorus and oxygen atoms as well as the canonical Simplified Molecular-Input Line-Entry System (SMILES) were calculated.

To avoid the time intensive manual assignment of multiple ^31^P shifts in molecules with more than one phosphorus atom, only molecules with exactly one phosphorus atom are listed in the database. This also excludes symmetric molecules, which feature only one ^31^P shift for multiple, but magnetically identical phosphorus atoms.

Subsequently, the SDF entries were transformed into molecular graphs using the R packages tidygraph [29], magrittr and dplyr [30]. The molecular graphs were used to calculate an easily human-readable character string representing the direct bonding partners of the phosphorus atom, called the environment label. E.g. the string “C1C1C1” represents a phosphorus atom bound to three carbon atoms each bound via a single bond to the P atom. Another example would be “O1O1O1O2” which represents a phosphate. This is comparable to HOSE codes [8] to provide an easy-to-understand, precomputed handle to filter the database.

Finally, the NMR information (shift, solvent, reference) is saved to the data block of the respective SDF file using the tags suggested by the NMReData initiative [31]. Additionally, the data is also available as CSV file featuring a unique ID, the sum formula, the molecular weight, the number of carbon, nitrogen, phosphorus and oxygen atoms, the canonical SMILES strings, the reference, the source as well as the custom environment label. The last available format is an R tibble [32], a variant of a data frame, which contains the same information as the CSV file as well as the SDF object and the molecular graph object for each molecule.

A complete manual check of all molecules, their structure and their ^31^P NMR shifts as reported in the original references is not possible. We performed a manual check on 142 randomly selected molecules, which is equivalent to 1% of the database and found no errors for these entries. This is also true for the references published after 1975, when the orientation of the ppm scale was inverted for ^31^P NMR spectroscopy. We thus assume that the original peer-review and control process for the two books from which the data was extracted was of good quality. We also used the spectrum prediction implemented in MestReNova (14.2.3-29241) [33] on the 142 manually checked molecules and found no major deviations. Smaller deviations between the entries may stem from different solvents, from slightly different experimental measurement conditions or that MestReNova’s prediction algorithm cannot predict the ^31^P signal of the desired structure due to a lack of training data. This does not fully exclude errors either in the original references, the book sources, or our digitalization process.

### NMR signal prediction

With the “Ilm-NMR-P31” database at hand, it becomes possible to compare the performance of four model groups for the prediction of ^31^P NMR shifts. These are namely (1) a HOSE code-based model, (2) linear increment models based on substituents at P atoms, (3) linear and non-linear models based on fingerprints, and (4) a GNN.

**Fig. 1:**
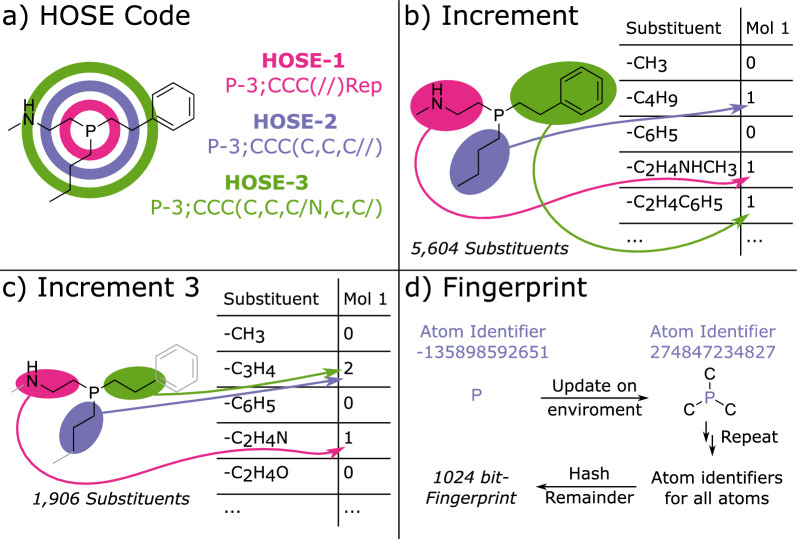
Different concepts how to represent molecules for NMR shift prediction. **a** HOSE codes look at the environment of a given atom, here P atom. **b** Increment models rely on the partition of the molecule into different substituents which are bound to the P atom. **c** Same as **b** but only considering atoms close to the P atom of interest. **d** Fingerprints look at different molecular fragments which might not have a connection to the P atom of interest

The dataset was preprocessed, all models were built and all figures were made using R (4.3.1) and its packages rcdk [34], rcdklibs [35], fingerprint [36], tidyverse [37], tidygraph [38], yardstick [39], igraph [40, 41], ChemmineR [26], ChemmineOB [27], broom [42], magrittr [43], caret [44] and reticulate [45]. reticulate was used to build and train the GNN in conjunction with Python 3.9.13 as well as the python packages tensorflow [46], keras [47], keras-tuner [48], rdkit [49], and spektral [50]. As model metrics the mean absolute error (MAE), the root mean squared error (RMSE) and the coefficient of determination (R^2^) are calculated for all models.

For the HOSE code-based model the HOSE codes with a depth ranging from 1 to 5 (see Fig. 1a) were calculated using the R implementation of the Chemical Development Kit (CDK) [34, 51]. For any given test molecule, the model looked through the HOSE codes stored in the training dataset. Firstly, the model looked for a match of the HOSE-5 codes. If more than one match was found the average of the training ^31^P shift was used as a prediction for the test molecule. When no match is found for the HOSE-5 code the model falls back to the HOSE-4 code. This continues until either a match is found in the training dataset or in case that even the HOSE-1 code yields no result the model is not able to return a prediction. The dataset was split into a training (60%) and a test dataset (20%) for HOSE code prediction.

For the increment model the substituents bond to the phosphorus atom were extracted by virtually breaking all P-R bonds connecting the phosphorus to the molecules (see Fig. 1b). This yields a variable number of substituents per molecule, e.g. a phosphine usually yields three substituents when the phosphorus is not part of a ring. In total 5640 different substituents were extracted from the 14,250 molecules. Like the Bag of Substitutes (BoS) approach presented by Gensch et al. each molecule is represented by the type and number of substituents bond to the phosphorus atom [13]. The result is therefore a 14,250 × 5640 matrix. The same substituent extraction procedure was repeated on a dataset in which the molecules were subset to only contain atoms which are at most 3 bonds away from the central phosphorus atom (BoS3, see Fig. 1c). The idea behind this approach was to reduce the number of substituents. Atoms or groups that are far away from the central phosphorus should not influence the ^31^P shift significantly. This reduction in complexity should thus improve the quality of the increment approach for predicting ^31^P shifts. Consequently, only 1906 individual substituents were found in the BoS3 case. During model training it was found that exotic substituents which are present less than 10-times are limiting the model performance. Thus, two reduced datasets were derived which only contained information on molecules made up of substituents that are found 10 or more times in the original dataset. This leads to the BoS-Red and BoS3-Red dataset which contained 5681 molecules and 243 substituents or 5681 molecules and 161 substituents, respectively. Finally, two subsets of the BoS and BoS3 datasets were derived, which only contained phosphines (BoS-C1C1C1 and BoS3-C1C1C1).

To avoid the occurrence of unknown substituents during prediction, the training dataset was designed so that each substituent is present at least once in the training dataset. After this condition is fulfilled the remaining entries in the training, validation and test datasets are assigned randomly until the desired split is reached. This semi-random splitting process leads to an overestimated performance of increment models compared to the other models. It is nevertheless justified as otherwise the model training becomes impossible due to the scarcity of the input matrices. A multiple linear regression (MLR) as well as a linear ridge regression (LRR) model were fitted on the increment BoS, BoS-Red, BoS3, and BoS3-Red datasets. For the BoS-C1C1C1 and BoS3-C1C1C1 datasets only a LRR model was fitted. The RMSE of the validation set was used for hyperparameter optimization. The hyperparameters α and λ found in the LRR model were optimized via 4-fold cross validation (repeated 3-times, dataset split: training/test 80%/20%) with the following hyperparameter ranges:

$${\text{LRR}}:\alpha = {10^{ - 10}} - 1;\lambda = 0 - 1.$$

Circular 1024-bit ECFP6 fingerprints (FPs) were calculated from the canonical SMILES strings using the R implementation of the CDK (see Fig. 1d) [34, 51]. Based on these FPs 5 different models were fitted, namely a MLR, a LRR, a random forest (RF), a k-nearest neighbor (KNN) as well as an extreme gradient tree boosting model (XGB). The RMSE of the validation set was used for hyperparameter optimization. The hyperparameters found in the different models were optimized via 4-fold cross validation (repeated 3-times, dataset split: training/test 80%/20%) with the following hyperparameter ranges:

$${\text{LRR}}:\alpha = {10{ - 10}} - 1;\lambda = 0 - 1.$$

$$\text{RF}:\text{mtry} = 3 - 1000;\text{splitrule} = "\text{variance}","\text{extratrees}";\text{min}.\text{node}.\text{size} = 5$$

$$\text{KNN}:{\text{k}}= 3-60$$

XGB: nrounds = 250–1500; max_depth = 3; η = 0.2–0.4; gamma = 2; colsample_bytree = 0.8; min_child_weight = 1; subsample = 0.5.

The GNN used a graph representation of the molecules as input. The atomic number, the number of bonding partners, the formal charge, the chirality, the number of hydrogen atoms attached, the hybridization, the atomic mass, and if the atom is part of an aromatic structure were used as atom features. The features were calculated using RDKit [49]. The GNN does not consider the influence of edge features and thus does not consider the nature of bonds between the atoms. The optimal GNN design identified by You et al. [52] as implemented in spektral was used as a basis [50]. In short, the GNN applies batch normalization, uses 2 pre-processing layers, 4 message passing layers (MPLs) with 256 hidden channels and sum aggregation in each layer, 2 post-processing layers, and a final global sum pooling layer. It skips connections with concatenation and uses the PReLU activation function. The molecular graphs were presented in batches of 32 via their disjoint union. The dataset was split into a training (60%), validation (20%), and test dataset (20%) for GNN training and evaluation. The mean squared error (MSE) of the validation set was used for hyperparameter optimization. A hyperparameter optimization was done on a random subset of 1.000 graphs (600 training, 200 validation). The following hyperparameter ranges were scanned for 1.000 epochs each:

GNN: Number of preprocessing layers = 1–2; Number of MPLs = 4, 6, 8; Number of postprocessing layers = 1–2; Learning rate = 0.001, 0.01.

The final model was trained for 3.000 epochs using the entire dataset. Peak performance was reached after 500 epochs.

## Results and discussion

### Ilm-NMR-P31 database

#### Database statistics

**Fig. 2:**
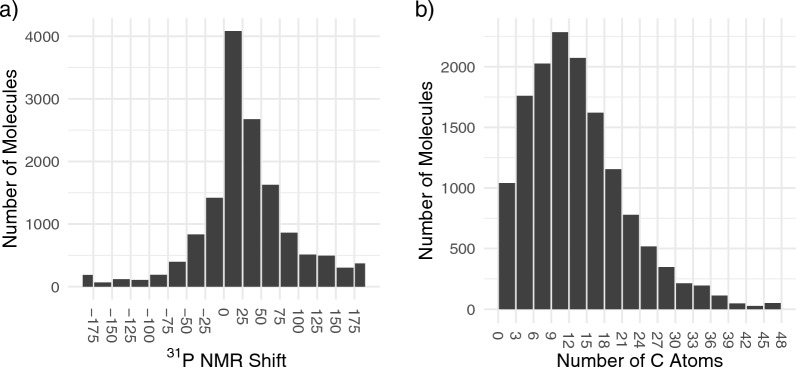
Distribution of ^31^P shifts (**a**) and of number of carbon atoms (**b**) in the database

Currently, the Ilm-NMR-P31 database lists 14,250 entries, for 13,730 unique molecules from 3,648 unique references. The median number of carbon atoms per molecule is 13, the median molecular weight is 305.63 g mol^−1^, while the median ^31^P NMR shift is 24.0 ppm (see Fig. 2 for distributions).

**Fig. 3:**
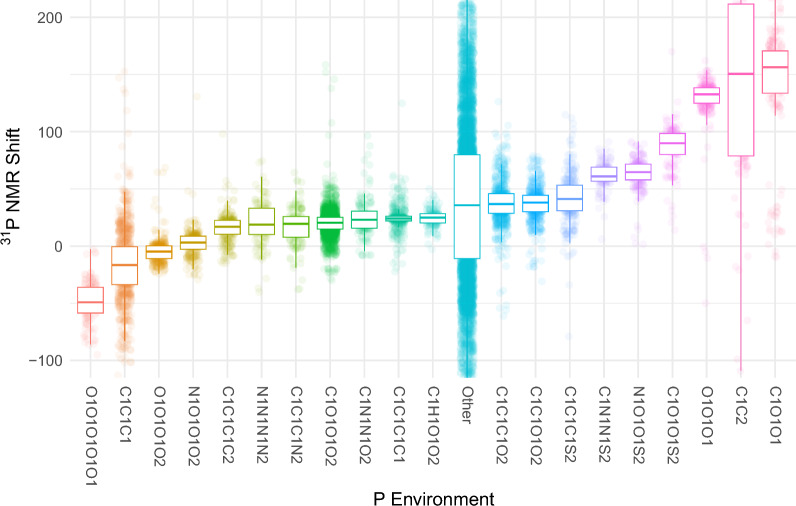
^31^P NMR shifts of the 19 largest subgroups by frequency. The remaining molecules are summarized under the “Other” label. Each point represents a single molecule while the box plots display the group median, the 25% and 75% quantile as well as the 1.5 interquartile range

The ^31^P NMR shift is mainly influenced by the direct binding partners of the phosphorus atom. Therefore, it is useful to divide the complete dataset into smaller subsets based on the molecular environment of the phosphorus atom. In total, 859 subsets can be derived in this way. Using this approach, the largest group of molecules are phosphonates (2351 entries) which feature a P = O, two P-O and a P-C bond. The second-largest group are phosphines (815 entries) where the phosphorus atom has three P-C bonds. The ten largest groups are summarized in Table 1 by their frequency, their median carbon atom count, their median molecular weight and their median ^31^P NMR shift. Many structure motives are only present a negligible number of times. Figure 3 depicts the ^31^P NMR shift of the 19 most common groups as box plots and summarizes the remaining compounds under the “Other” label. The 19 most common groups account for 7104 molecules, which represent 49.9% of the entire dataset.

**Table 1:** Ten of the largest subgroups by the direct environment of the phosphorus atom found in the Ilm-NMR-P31 database and their frequency, the median number of carbon atoms, the median molecular weight, the median ^31^P NMR shift as well as the standard deviation of the ^31^P shifts per subgroup

P Env.	Freq.	Med. C Atoms	Med. MW	Med. ^31^P Shift / ppm	SD ^31^P Shift / ppm
C1O1O1O2	2351	13	309.3	20.4	11.7
C1C1C1	815	16	289.4	− 16.5	42.0
C1C1C1O2	606	16	305.1	36.7	19.9
C1C1O1O2	428	13	283.5	38.1	15.7
C1C1C1C1	375	28	499.5	24.1	22.7
O1O1O1O2	312	13	313.8	− 4.9	22.9
C1C1C1C2	275	27	442.4	16.9	12.4
N1O1O1O2	249	12	298.3	3.0	13.3
C1C1C1S2	245	15	298.4	41.2	22.7
O1O1O1	208	9	226.9	132.7	29.6

It comes to no surprise that the ^31^P NMR shifts mainly lay between − 200 and 200 ppm as it is commonly known in analytical chemistry [53]. To be precise 95% of all ^31^P shifts from the database lay between − 127.5 and 176.5 ppm. The group of molecules with the largest ^31^P NMR shift from which at least 10 molecules are present in the database are phosphonium cations with two hydrogen atoms, one P-N and one P-S bond (median ^31^P shift: 415 ppm), while the group with the smallest ^31^P shift are primary phosphines where the organic residual is bond via a Si atom to the phosphorus (median ^31^P shift: − 238.5 ppm). The most structure-insensitive group are molecules from the C1H1N1O1O1 subgroup as their ^31^P NMR shift standard deviation for the group is only 4.9 ppm for 10 molecules in the database.

#### Comparison to other NMR databases

NMR databases are commonly available for ^1^H and ^13^C nuclei but are limited for other nuclei like ^31^P, ^19^F or ^11^B. To the best of our knowledge, the largest database available for ^31^P NMR spectra is Wiley’s KnowItAll™ database [54] which features 14,375 compounds with one or more phosphorus atoms. Besides their own database Wiley also partnered with Wolfgang Robien who collected and curated extensive number of NMR spectra [55]. The ^31^P database of Wolfgang Robien contains 23.180 molecules. The largest disadvantage of these two databases is that they are not open access.

In comparison the Ilm-NMR-P31 database has roughly the same size (14,250 entries) as the two commercially available databases from Wiley. The scope of our database is currently limited to molecules with only one phosphorus atom, which is not the case for the two other databases. Other commonly used open access NMR databases like NMRshiftdb2 [56, 57], the NIST WebBook [58], the Biological Magnetic Resonance Data Bank [59] or the SDBS [60] database only contain a small amount of ^31^P data or none at all. Our database thus covers an important gap between large, commercial and small, open-access ^31^P databases.

#### Database limitations and future development

The greatest limitations of our database at the moment are the lack of components with more than one phosphorus atom and the absence of coupling constants. A minor shortcoming is that no molecules younger than 2014 are included in the database.

In the future, the database shall be extended in three ways: Firstly, more ^31^P data should be added to the database. The shortcoming of digitalizing table data is the static nature of the data included in the original books. Therefore, the database in its version 1.0 only includes data up until 2014. New data should ideally be added automatically. Our vision to achieve this goal is to leverage the increasing number of open access articles and automatically scrap ^31^P information from newly published research articles. Many publishers like Wiley, the American Chemical Society (ACS), the Royal Society of Chemistry (RSC), Springer-Nature and Elsevier offer access to articles published by them for large-scale data collections.

Secondly, the broadness of the database should be improved by adding molecules with more than one phosphorus atom to the database. At the moment, the assignment is easy as one ^31^P shift can be assigned to one molecule. If more than one phosphorus atom is present in a molecule, the shifts need to be assigned to the individual atoms. In terms of graph theory this means that the database must switch from a graph-based assignment to a node-based assignment. This is not new as NMR shift assignment to individual atoms is also necessary for ^1^H and ^13^C spectra. Thus, the NMReData initiative already defined a format which can be used to represent the per-atom assignment. The challenge that remains is the correct assignment of the ^31^P shifts to the individual atoms. A solution might be to train a CASE system on the present database and use its automatic signal assignments to relief the burden of individual, human-made assignments.

Thirdly, information about coupling constants should be added to the database. The information of coupling constants is less often found in research articles, but nevertheless provides important information for structure elucidation and component identification. For the inclusion of coupling constants, the database needs to move to node-based assignment. As described earlier, the NMReData format provides a framework on how this information can be reported in a digital format.

### ^31^P NMR signal prediction

The main use of the database will most likely be the training of signal prediction and CASE systems. ^31^P nuclei are rarely included in these systems at the moment. A noteworthy exception is Mnova’s MestReNova [33] software which offers a prediction plug-in called NMRPredict which can also predict ^31^P NMR spectra. It uses a Hierarchically Ordered Spherical Environment (HOSE) code- as well as a neural network-based prediction algorithm. Another example is the report from Liu et al. who used a multiple linear regression model based on atomic ionicity indices and stereoscopic effect parameters to predict ^31^P NMR shifts of 291 phosphines [15].

**Fig. 4:**
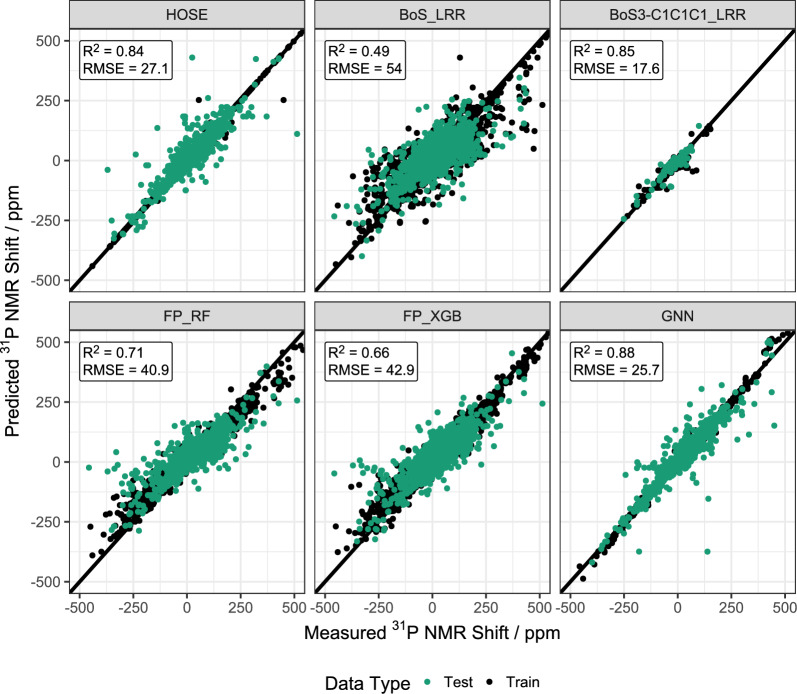
Parity plots showing the ^31^P NMR shift prediction performance for different models

#### HOSE code model

As discussed in numerous publications, HOSE codes are a simple yet surprisingly effective way to predict NMR signals. HOSE codes were therefore tested to predict ^31^P NMR signals. The resulting parity plot can be seen in Fig. 4 while the MAE, RMSE and R^2^ values can been found in Table 2. With a MAE of 11.9 ppm over a range of 500 ppm HOSE code-based prediction performs very well. This regression quality is on par with ^31^P shifts calculated by quantum chemical methods [4]. The largest deviations are found for substances whose HOSE codes are not well represented in the training dataset and thus only lower quality HOSE-1 or HOSE-2 codes are used for prediction. In addition, it was found that the prediction errors were not linked to a specific class of molecules.

#### Increment models

Encouraged by the promising results of the HOSE code-based prediction an increment-based model was derived. The assumption for an increment model is that the substituents bound to the phosphorus atom contribute linearly to the ^31^P shift:

$${\delta }_{31P}=b+\sum _{i}{a}_{i}\cdot {n}_{i}$$

where *δ*_*31P*_ is the predicted ^31^P NMR shift, *b* a constant offset in the MLP model, *a*_*i*_ the coefficients describing how much a given substituent *i* influences the ^31^P shift and *n*_*i*_ the number of substituents *i* found in a molecule of interest. The advantage of an increment model is that the coefficients *a*_*i*_ can be interpreted as how much electron density a given substituent contributes or withdraws from the P atom as the electron density around a nucleus is directly linked to its shielding constants and thus its NMR shift. The coefficients *a*_*i*_ therefore have a physical meaning which is not the case for HOSE codes and only partially true for FPs. Gensch et al. used this approach successfully to predict quantum chemical descriptors for phosphorus containing components [13]. They named the approach Bag of Substituents (BoS) after the similar technique Bag of Words used in language processing. Unfortunately, neither a multi linear regression model (LMR) nor a ridge regression model (LRR) performed well (MAE: 35.1 and 35.8 ppm, see Fig. 4).

One idea to explain why the linear BoS models did not perform well was the large number of unique substituents found in the dataset. 14.250 molecules yielded 5.640 substituents, off which 3.517 substituents were only present in one molecule. To circumvent this problem, the molecules were reduced to only contain atoms which are at most three bonds away from the central phosphorus atom (BoS3). The reasoning for this approach is that atoms that are far away from the phosphorus do not influence the electron density at the atom of interest. This idea thus is a crossover of the increment and the HOSE code approach. In this reduced dataset only 1.906 substituents were found. The MAE for the LM as well as the LRR improved slightly to 29.9 and 32.1 ppm but is still worse than the HOSE code-based model.

A second idea to improve the performance of the increment model was to remove molecules with rare substituents (frequency < 10 occurrences) from the dataset (BoS-Red and BoS3Red). This dataset with presumedly improved quality unfortunately also did not improve the prediction performance (see Table 2). A key finding from the failure of the increment models is that the substituents interact with each other in a non-linear manner when modeling the electron density at phosphorus atoms.

Finally, we were interested if the performance of the increment model improved when it was only trained on a specific class of phosphorus containing molecules. Encouraged by the work of Tong et al. who successfully modelled ^31^P NMR shifts of phosphines using ionicity indices we decided to test this hypothesis on phosphines (BoS_C1C1C1 and BoS3_C1C1C1). It was found that with a LLR model the MAE dropped significantly and for BoS3_C1C1C1 a good value of 10.7 ppm was reached surpassing the HOSE code-based model for this class of molecules (see Fig. 4). This finding shows that increment-based models can work in the prediction of ^31^P NMR shifts with the restriction that the model has to build for each class of molecules, e.g. phosphines or phosphates, separately. As the size of the dataset is reduced, a much larger starting dataset is needed to train accurate models for each molecule class.

Coming back to the physical interpretability of the BoS3_C1C1C1 model, different coefficients *a*_*i*_ were found. As expected, e.g. methyl groups push electron density onto the phosphorus atom with *a*_*CH3*_ = - 7.52 ppm/group, while pentafluoro ethyl groups withdraw electron density at *a*_*C2F5*_ = + 10.08 ppm/group.

**Table 2:** Mean Absolute Error (MAE), Root Mean Squared Error (RMSE) and Coefficient of Determination (R^2^) for different models during ^31^P NMR shift prediction

Prediction Method	MAE/ppm	RMSE/ppm	R^2^
HOSE	11.9	27.1	0.84
BoS_LMR	35.1	57.3	0.48
BoS_LRR	35.8	54.0	0.49
BoS3_LMR	29.9	55.6	0.57
BoS3_LRR	32.1	55.6	0.50
BoS-Red_LMR	42.3	59.7	0.50
BoS-Red_LRR	42.2	59.7	0.50
BoS3-Red_LMR	38.1	55.6	0.49
BoS3-Red_LRR	38.1	55.6	0.49
BoS-C1C1C1_LLR	16.8	26.2	0.63
BoS3-C1C1C1_LLR	10.7	17.6	0.85
FP_LMR	41.1	59.8	0.35
FP_LRR	39.3	59.3	0.35
FP_KNN	28.6	56.5	0.48
FP_RF	20.9	40.9	0.71
FP_XGB	27.0	42.9	0.66
GNN	11.4	25.7	0.88

#### Fingerprint models

The analysis of the increment models revealed that in general the ^31^P NMR shift is influenced in a non-linear way by the substituents bound to the phosphorus atom. A way to capture exotic functional groups and non-linear interactions are FP based models. In short, the underlying technique used to calculate circular FPs which are applied in the following analysis is similar to how HOSE codes are designed. The environment around each heavy atom is analyzed and then folded into a vector of specific length, 1024 bits in this study. We therefore expected that circular FPs better represent the molecular features that contribute to ^31^P NMR shifts.

In total, five different models were trained on the circular FPs. The linear models LMR and LRR did not perform well with MAE of 41.1 and 39.3 ppm (see Table 2). This was expected given the results from the increment models, although it was surprising to see that the FP-based models performed worse than the increment models when the same base model was used. An explanation might be that the folding of the molecular information into 1024-bit FPs is too condensed for linear models to perform successfully. A k-Nearest Neighbor (KNN) approach reduced the MAE to 28.6 ppm but still did not yield satisfying prediction performance.

Reasonable prediction performance was achieved by a Random Forest (RF) and an Extreme Gradient Boosting (XGB) model, also based on decision trees, with MAEs of 20.9 and 27.0 ppm, respectively. The regression based on FPs and an ensemble of decision trees thus captures the non-linear essence of the ^31^P NMR shift formation, but still does not achieve the same performance as the HOSE code-based model. It is difficult to give a precise physical explanation for this finding as the FPs act as a black box and thus the interpretation of the structure of the RF or XGB models is impossible.

#### Graph neural network

As Jonas and Kuhn detailed in their review [9], GNNs are currently the best performing models for the prediction of ^1^H and ^13^C NMR shifts. Naturally, a GNN was also tested for the prediction of ^31^P NMR shifts. The GNN was based on the optimal GNN structure found by You et al. who tested a wide range of hyperparameters, network layouts and analysis tasks [52]. A hyperparameter optimization performed on a subset of 1000 randomly selected molecules revealed that the ideal GNN for the prediction of ^31^P NMR shifts should consist of 6 MPLs and only 1 preprocessing layer as opposed to the layout found by You et al. which utilized 4 MPLs and 2 preprocessing layers. Besides these minor deviations, the GNN trained for the prediction of ^31^P shifts is identical to the general model described by You et al.

As can be seen from Fig. 4; Table 2 the GNN performed very well with an MAE of 11.4 ppm. The GNN performs even better than the HOSE code model and both models perform on the same level as quantum chemical calculations [4] but are significantly faster.

**Fig. 5:**
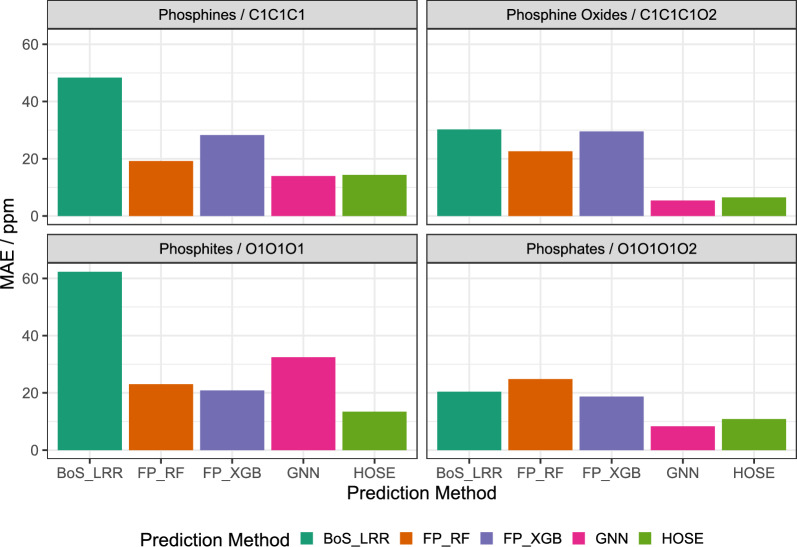
Bar plots showing the Mean Absolute Error (MAE) during ^31^P NMR shift prediction performance for different models when looking at specific classes of phosphorus containing molecules

The model comparison revealed that HOSE code and GNN-based models perform the best and on par with quantum chemical calculations when predicting ^31^P shifts. In general, increment and circular FP based models perform worse, but it was found that in special cases like the prediction on a specific class of phosphorus containing molecules linear increment models perform well and have the additional advantage of physical interpretability. Figure 5 analyzes the MAE of different models when looking at special classes of phosphorus containing molecules. The model performances follow the general trend for the complete dataset shown in Fig. 4. Interestingly, the MAE is not homogeneous for the same model across different molecule classes. E.g. the GNN performs significantly worse for phosphites even to the extent that FP_RF, FP_XGB and HOSE code based models are all superior for this specific class of molecules. This finding might be the reason why the commercial prediction software offered by MNova or ACDLabs use an ensemble-based prediction which combines HOSE code and GNN-based models. Our study shows that it might be useful to incorporate other models like FP-based RF prediction into the ensemble technique as well.

## Conclusions

In conclusion, this article presented the new ^31^P NMR shift database Ilm-NMR-P31 that fills the gap between commercially available databases and limited open-access resources. The database currently contains 14,250 entries, covering 13,730 unique molecules from 3,648 references. It provides valuable information on the structure and ^31^P NMR shift of organic and inorganic compounds. The database is a valuable resource for data mining and machine learning applications, particularly for the training of signal prediction and Computer-Assisted Structure Elucidation (CASE) systems. Additionally, the article presented and discussed multiple approaches for the prediction of ^31^P NMR shifts. It was found that models based on a graph neural network or on hierarchically ordered spherical environment codes perform the best with mean absolute errors of 11.9 and 11.4 ppm, respectively. In addition, linear increment models focusing on a specific class of phosphorus containing molecules also yield good results, albeit in a much more confined molecular parameter space. Overall, this new ^31^P NMR shift database provides an important open-access resource, enabling advancements in NMR spectroscopy, signal prediction and structure elucidation.

## Data Availability

The data is available online at the GitHub repository https://github.com/clacor/Ilm-NMR-P31. The dataset is also available from Zenodo 10.5281/zenodo.8260783. The R files used for the model analysis are available as Additional Files. The relevant graph processing R scripts were published as an independent R package available from GitHub https://github.com/clacor/MolGraphR.

## Abbreviations

ACS: American Chemical Society
BoS: Bag of substituents
CASE: Computer–assisted structure elucidation
CDK: Chemistry development kit
DFT: Density functional theory
FP: Fingerprint
GIAO: Gauge–independent atomic orbital
GNN: Graph neural network
HOSE: Hierarchically ordered spherical environment
KNN: k–Nearest Neighbor
LMR: Multiple linear regression
LRR: Linear ridge regression
MAE: Mean absolute error
ML: Machine learning
MPL: Message passing layer
MSE: Mean squared error
NMR: Nuclear magnetic resonance
OCR: Optical character recognition
PReLU: Parametric rectified linear unit
RF: Random forest
RMSE: Root mean squared error
RSC: Royal society of chemistry
SDBS: Spectral database for organic compounds
SMILES: Simplified molecular–input line–entry system
XGB: Extreme gradient boosting
